# Does the Mediterranean Dietary Pattern Promote Lower Sodium Urinary Excretion in Children?

**DOI:** 10.3390/children10091478

**Published:** 2023-08-30

**Authors:** Mónica Rodrigues, Francisca de Castro Mendes, Patrícia Padrão, Luís Delgado, Renata Barros, João Cavaleiro Rufo, Diana Silva, André Moreira, Pedro Moreira

**Affiliations:** 1 Faculty of Nutrition and Food Sciences, University of Porto, 4150-180 Porto, Portugal; carolina101997@hotmail.com (M.R.); patriciapadrao@fcna.up.pt (P.P.); renatabarros@fcna.up.pt (R.B.); andremoreira@med.up.pt (A.M.); 2 Basic and Clinical Immunology, Department of Pathology, Faculty of Medicine, University of Porto, 4200-319 Porto, Portugal; francisca_castromendes@hotmail.com (F.d.C.M.); ldelgado@med.up.pt (L.D.); disolha@gmail.com (D.S.); 3 Epidemiology Research Unit, Laboratory for Integrative and Translational Research in Population Health, Institute of Public Health, University of Porto, 4050-600 Porto, Portugal; 4 Immuno-Allergology Department, Centro Hospitalar São João, 4200-319 Porto, Portugal; 5 Center for Health Technology and Services Research (CINTESIS@RISE), Faculty of Medicine, University of Porto, 4200-319 Porto, Portugal

**Keywords:** Mediterranean diet, sodium, children, urinary sodium excretion

## Abstract

An adequate sodium intake is related to various health benefits. Parallelly, the Mediterranean diet (MD) is a dietary pattern known for its many positive impacts on health. Nonetheless, the association between adherence to the MD and sodium urinary excretion is scarce, even more in children. This study aimed to assess the association between MD adherence and the excretion of sodium, as a proxy of intake. This cross-sectional analysis comprised 295 children (46.8% females, aged 7–11 years, mean age: 8.53 ± 0.73 years) from 20 schools within Porto, Portugal. MD adherence was evaluated utilizing the alternate Mediterranean score (aMED). Higher scores denote a healthier dietary pattern (0–8). Sodium excretion was estimated by 24-h urine collection. The association between adherence to MD and Na excretion was estimated by logistic regression, adjusting for confounders. Children in the higher sodium excretion group had a higher intake of legumes, a higher body mass index and parents with lower education levels compared to children in the lower sodium excretion group. In logistic regression analysis, sodium urinary excretion was not associated with higher MD adherence, even after adjustment for confounders. High MD adherence could not be associated with lower sodium excretion in children.

## 1. Introduction

Studies conducted in various countries have consistently shown that children and adolescents tend to consume more sodium than the recommended levels [1].

Excessive sodium intake can have adverse effects on health, particularly concerning cardiovascular health [2]. High sodium intake has been connected to an elevated risk of hypertension as well as stroke and heart disease later in life. [2]. Based on the latest epidemiological research, the occurrence of childhood arterial hypertension in 2015 was approximately 4.32% in 6-year-olds, 3.28% in 9-year-olds, and reached its highest value at 7.89% in 14-year-olds [3]. 

The guidance provided by the World Health Organization (WHO) suggests that limiting the consumption of sodium is beneficial for managing blood pressure in children. The WHO advises that adults should aim for a maximum daily sodium intake of 2 g. This value is recommended to be adapted for children, taking into account their energy needs in comparison to those of adults [4]. The European Food Safety Authority (EFSA) Panel on Nutrition also suggests that the safe and appropriate sodium intake levels for children may be estimated based on the values recommended for adults, taking into account the children’s energy needs and growth. The recommended daily sodium intakes are as follows: for children aged 1 to 3 years, the suggested amount is 1100 mg per day. For those between the ages of 4 and 6 years, the recommended intake is 1300 mg daily; children aged 7 to 10 years are advised to consume around 1700 mg of sodium per day; lastly, for adolescents aged 11 to 17 years, the recommended daily sodium intake is 2000 mg. [5].

Dietary habits, such as the preference for high-sodium foods, may be established in childhood [2], and consistently following a dietary pattern characterized by high sodium, energy-dense foods, excessive fat, refined carbohydrates, added sugar, and inadequate intake of fruits and vegetables significantly elevates the risk of developing hypertension and cardiovascular disease [6]. The latest dietary guidelines have placed greater emphasis on considering overall dietary patterns and their impact on blood pressure [6]. Among the dietary patterns, the Mediterranean diet (MD) has been notably recognized [6,7]. 

The Mediterranean dietary pattern is traditionally described as mostly plant-based, low in saturated fat and red meat, and high in fruits and vegetables, whole grains, and nuts [8,9]. This dietary pattern has been related to many beneficial health outcomes [7,9,10,11,12]. Most of the advantages of the MD pattern arise from its polyphenol, vitamin, mineral, and fiber-rich content [13] that results in antioxidant and anti-inflammatory properties [14]. Additionally, the synergistic effects of the combination of the types of foods and nutrients from a whole dietary pattern also account for its health benefits [14]. Nonetheless, food habits are evolving, and food processing and new types of foods are being introduced into our dietary patterns [8]. Taking this into consideration, some foods that are (ultra)processed, energy-dense, and salt-rich may infiltrate their way into the MD [15].

The impact of salt within the context of this dietary pattern remains uncertain. While it is assumed that sodium intake would be appropriate in the Mediterranean dietary pattern, some studies have examined the link between adherence to this diet and sodium consumption, yielding inconsistent findings [16,17,18,19,20].

A study reported that mean urinary Na^+^ concentrations were significantly decreased in participants in the highest tertiles of adherence to the MD [16]. A different study found that there was no statistically significant difference between the lowest and highest quartiles of MD adherence regarding salt intake, as estimated by sodium urinary excretion [17]. Nonetheless, it was revealed that sodium intake was increased in children with moderate/high adherence to the Mediterranean diet [18]. Kanauchi et al. [19], in a study with Japanese adults, also found that higher adherence to the MD pattern was linked to increased salt intake. Additionally, more adherence to the Mediterranean diet did not show an opposite relationship with hypertension when considering sodium and potassium intake, implying that lower sodium consumption might be a mediating element for the effects of the Mediterranean diet on hypertension [20]. 

Despite numerous positive associations between the MD and health, this dietary pattern could serve as a font of concealed sodium, arising from mutually added salt throughout cooking or at the table, as well as the inclusion of products, such as bread and cheese, containing significant amounts of salt [18]. 

The aim of this study was to quantify sodium excretion by 24 h urine collection and assess its association with adherence to a Mediterranean dietary pattern in a sample of children.

## 2. Methods & Materials

### 2.1. Participants and Study Design

**Figure 1:**
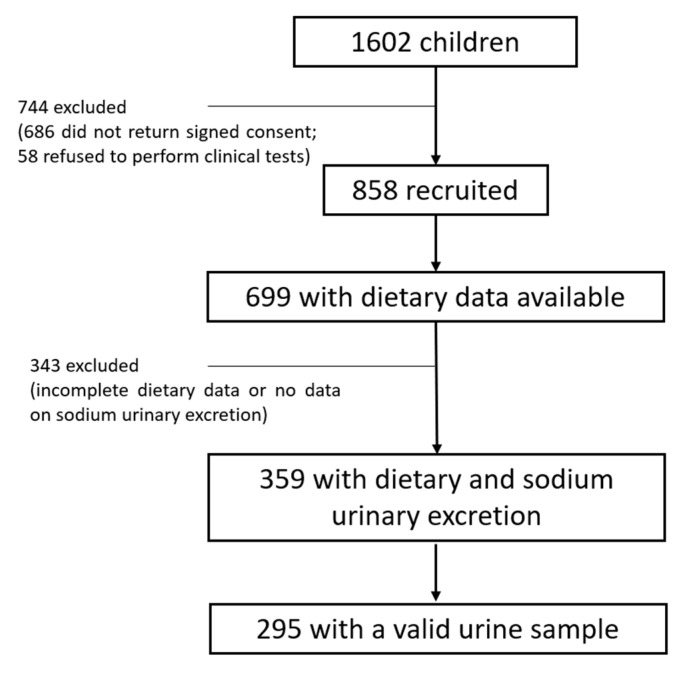
Flow chart of the included participants.

This is part of a cross-sectional analysis which took place in Porto, Portugal, from 2014 to 2015. The original study included 1602 children between the ages of 7–12 years old who were enrolled in the 3rd and 4th grades at 20 public schools [21]. Of these children, 58 (3.8%) declined to undergo clinical procedures and 686 (42.8%) did not provide signed informed consent. Among the continuing 858 children (53.6%), 359 (41.8%) had simultaneously full nutritional information regarding to aMED score and data on sodium urinary excretion, and 295 (82.2%) were included in the analysis (Figure 1). From the legal caretaker of each children, written consent was acquired, and the investigation was conducted in accordance with the Helsinki Declaration. The Ethics Committee of the University Hospital São João approved the study (ARIA 248-13).

### 2.2. Participants Assessment

#### 2.2.1. Diet Quality and Dietary Evaluation

In order to collect dietary information, a trained interviewer administered a single 24 h food recall questionnaire to the children. The questionnaire followed well-established procedures and utilized a photograph atlas to help make an estimation of portion sizes accurately. Detailed inquiries were made about the children’s beverage and food and consumption over the previous 24 h, including detailed information such as products brands and quantities of the recalled intake [22]. Resourcing to the software Food Processor^®^, made by ESHA Research in the United States, SQL^®^, V3, nutritional data and total energy intake (measured in kilocalories) were computed. This program integrates databases that include detailed data on the Portuguese foods nutritional composition.

The level of adherence to the traditional Mediterranean diet was evaluated through the alternate Mediterranean diet (aMED) score [23]. The aMED score is an adapted version of the original Mediterranean diet scale [11]. Even though the initial score accounts for ethanol/alcohol, as children do not consume this component, this element was eliminated from the scoring system..To assess adherence to the Mediterranean diet, the total aMED score was calculated, ranging from zero to eight points, where higher scores indicated healthier diets.

The aMED score was determined based on the consumption of eight specific food items: red and processed meats, the ratio of monounsaturated to saturated fat (MUFA:SFA), vegetables, fresh fruits, nuts, whole grains, fish, and pulses. and Each of these components was assigned either one or zero points, depending on whether the participant’s intake was equal to or above the sex-specific median (one point) or below the median (zero points), excluding processed and red meats, where consumption below the median earned one point.

#### 2.2.2. Urine Collection—24 h

Caretakers or parents were provided with verbal and written directions together on how to assist children in collecting a 24 h urine sample. A typical sterilized urine collection bottle for this purpose was given. On the first morning of the collection, they were instructed to discard the initial specimen and then proceed to collect all subsequent specimens for a continuous 24 h period, including the first specimen of the following day. The collected samples were sent to certified laboratories for analysis, where they were tested for 24 h creatinine (mg/day), 24 h urine volume (mL), and 24 h sodium (mEq/day). To standardize the measurements, urinary sodium values were converted to mg/day (where 23 mg Na^+^ = 1 mmol Na^+^ or 1 mEq Na^+^) [24]. To assess completeness of the 24-h urine collections, creatinine coefficients as creatinine excretion in relation to weight were used. Creatinine coefficients above of 0.1 mmol·kg^−1^·day^−1^ were classified as an acceptable 24-h urine collection [25]. 

Additionally, as urine collection is the ‘gold standard’ method for estimating salt intake, to estimate sodium consumption, we assumed that approximately 90% of consumed sodium is excreted in the urine [26]. 

#### 2.2.3. Covariates

Body mass index (BMI) was calculated by dividing the weight by the square of height, expressed in kilograms per square meter (kg/m^2^). Participants’ body weight was determined using a Tanita™ BC-418 Segmental Body Analyzer (Middlesex, UK), a digital scale, the obtained weight was documented in kilograms. Standing height was measured using a portable stadiometer (SECA^®^ 214) and recorded in centimeters. Both were measured with the child upright and standing without shoes. Later, participants were categorized into two groups: non-overweight/non-obese (below the 85th percentile) and overweight/obese (equal to or above the 85th percentile) based on BMI percentiles, considering specific sex and age percentiles as outlined by the US Centers for Disease Control and Prevention (CDC) [27].

Parental education was considered and categorized into three groups: ≤9 years; ≥10 to ≤12 years; and >12 years of regular school education [28].

Physical activity levels were assessed through a questionnaire distributed to each parent or caretaker. The questionnaire inquired about participation in sports activities outside of school physical education classes and were defined on three categories based on <2 times/week, 2–3 times/week, or >4 times/week.

#### 2.2.4. Statistical Analyses 

The statistical analyses were performed using the software SPSS^®^ statistical package version 27.0. In order to evaluate the normality of the continuous variables of our study, the skewness and kurtosis test was utilized. Participant characteristics were presented as percentages for categorical variables for the whole sample, while non-normally distributed continuous variables were expressed as median (25th–75th percentile), and normally distributed continuous variables were presented as mean ± standard deviation (SD).

To investigate the differences between children above or below the median for urinary sodium excretion, independent-samples *t*-tests were applied for continuous variables, and chi-squared tests were used for categorical variables. In cases of non-normal distribution, the Mann–Whitney test was used for inferential analysis.

To assess the associations between the adherence to the MD by the aMED score and the urinary sodium excretion of our sample, logistic regression models (odds ratios (OR), 95% confidence intervals (CI)) were utilized.

The significant differences across our study were determined at an α-value of less than 5%, corresponding to a 95% confidence interval (*p* < 0.05).

## 3. Results

Children had a median (25th–75th) sodium urinary excretion of 2484 mg (1886 mg–3197 mg) and approximately a median (25th–75th) intake of 2235 mg (1697 mg–2877 mg), as estimated by the 24 h sodium urinary excretion. 

The characteristics of the participants included in the study are presented by the total of the participants as well as by the lower sodium urinary excretion group (≤2484 mg) and the higher sodium urinary excretion group (>2484 mg) in Table 1.

The mean age of children was 8.53 (±0.73) years, and 46.8% (*n* = 138) were girls. The prevalence of overweight or obese children (*p* ≥ 85th) was 25.3% (*n* = 91) and the mean ± SD of aMED score was 2.66 ± 1.54. There are no significant differences between children in the ≤2484 mg 24 h urinary sodium excretion group compared with the children in >2484 mg group, except for age (8.45 ± 0.63 y vs. 8.63 ± 0.82 y), sex (females: 55.0% vs. 38.4%), BMI (overweight/obese: 17.4% vs. 29.4%), legume intake (0.00 g (0.00–0.00) vs. 0.00 g (0.00–33.38)), and TEI (2154.17 kcal (1817.75–2410.48) vs. 2224.12 kcal (1923.87–2559.93)). 

No significant association was detected between MD adherence and levels of urinary sodium excretion in crude model (OR = 0.96, IC95% 0.83–1.12). Even after adjustment for age, sex, parental education, total energy intake, physical activity, and body mass index, the aMED score did not have significant association with levels of urinary sodium excretion (aOR = 1.08, IC95% 0.91–1.28). Results are presented in Table 2. 

## 4. Discussion

This research unveiled that children from our study have a median urinary sodium excretion of 2484 mg and an estimated median intake of sodium of 2235 mg, which is above EFSA and WHO recommendations for children [4,5]. Moreover, children with higher adherence to the Mediterranean diet do not have a lower 24 h urinary sodium excretion, hence a lower sodium intake. 

These findings suggest that despite the Mediterranean diet being widely recognized for its health benefits, it does not appear to have a significant association with the amount of sodium consumed. Our study goes in agreement with the results by Viroli et al., where a high adherence to the MD was not associated with a different level of sodium intake compared with low adherence to the MD [29]. Accordingly, research conducted in Greece, the SING study, found no association between the intake of sodium, estimated through a 24 h urine excretion, and the adherence to Mediterranean diet [17].

Additionally, in children, Magriplis et al. found that a rise of 1 unit in the KIDMED score was linked to an increased likelihood of consuming sodium intake above the median value for most foods. These findings remained significant even after accounting for age, sex, BMI, and physical activity in the analysis [18]. On the other side, results presented by Serra-Majem et al., a study conducted among a group of Spanish adults, demonstrated that a reduction in sodium intake was observed among higher quintiles of adherence to the Mediterranean diet [30]. Nonetheless, the researchers emphasized that this research only encompassed individuals with a high level of education, and this group is typically associated with adopting healthier dietary patterns [30]. Moreover, it is essential to note that in the two previous referred studies [18,30], the assessment of sodium intake relied on a food frequency questionnaire, whereas in the current research, 24 h urinary sodium excretion was utilized, which is deemed the gold standard for evaluating sodium intake [31]. 

A nonexistent relationship between a lower sodium intake and adherence to the Mediterranean dietary pattern may seem surprising, as the MD has been linked to a reduced risk of hypertension, and in turn, hypertension is associated with excessive sodium consumption [32,33]. Conversely, a recent study carried out in a Mediterranean cohort aimed to investigate the connection between MD adherence and the development of hypertension [20]. Even though they initially found an inverse association between these two variables, this inverse association lost its statistical significance after considering sodium and potassium intake in the analysis [20]. This suggests that salt could be a crucial factor that mediates the effects of the Mediterranean diet concerning its impact on diseases such as hypertension. 

Furthermore, children from our study appears to have a low aMED score, this may be due to their insufficient consumption of nuts, whole grains, legumes and fish. 

Additionally, in our study, boys, overweight/obese children, and children with higher TEI%, were most likely to belong to the higher urinary sodium excretion group. 

It is referred, in other research, that boys usually tend to have a higher intake of sodium compared to girls, due to their overall higher energy caloric intake [34]. Moreover, in the study by Ma et al. [35], it has been established that the introduction of an extra gram of salt intake per day exhibits a positive association with having elevated odds, more precisely 28%, of a child falling into the category of having a BMI that is categorized as obese [35]. In the same previously referred research, it has observed that higher salt consumption was also significantly associated with total higher body fat mass in both adults (*p* = 0.001) and children (*p* = 0.001) after adjustment for age, ethnic group, sex, and energy intake [35].

Our study also found that children in the higher sodium urinary excretion group have a higher intake of legumes. Even though we do not have information on the nature of legumes (canned, fresh, dry, or frozen) we can speculate that this result could be due to the high content of sodium in canned legumes [36]. 

The concept of the Mediterranean diet was first explored by Ancel Keys in 1952 when he observed a lower incidence of cardiovascular disease in the Mediterranean region [8,37]. The Mediterranean dietary pattern was initially described as one characterized by low saturated fat intake and high unsaturated vegetable oil, plant-based food, and whole grain consumption [37], overall food groups naturally comprising low amounts of sodium [8].

In the following decades, as Mediterranean countries experienced economic and cultural changes, dietary patterns began to shift and the definition of the Mediterranean diet, as introduced by Keys, evolved to accommodate these variations [8]. Diverse definitions emerged, and different scores and methods to evaluate adherence to the Mediterranean diet appeared [13,38].

Modern societies are witnessing an increased consumption of processed and ultra processed foods, which often contain excessive amounts of sodium. These processed foods have gradually replaced traditional dietary components [15,23], leading to concerns about the potential impact on health [8].

While the original Mediterranean diet was associated with lower sodium intake due to its emphasis on natural and minimally processed foods, the present-day Mediterranean diet may not be as low in sodium [8,39,40]. The incorporation of processed foods into daily eating habits may have led to a rise in sodium consumption [18], and this may potentially offset some of the health benefits attributed to the traditional Mediterranean diet [20].

The results observed in our study may be a consequence of the gradual changing of culinary and food habits that have been observed in the last decades: an excessive inclusion of salt during cooking, adding too much table salt during meals, and/or more processed and ultraprocessed food intake. Even though children from our study with higher aMED scores may have a higher consumption of vegetables, fruits, legumes, whole grains, and healthy fats, they may parallelly have dietary habits that result in a high sodium intake. Processed vegetables, cheese, processed fish, cereals and cereal products, canned legumes, and canned tuna are all sources of salt [4], and are simultaneously components of our used index, the aMED score [23]. 

Our study has some limitations, one of them being that there was a single 24 h urine collection, which may not represent the individual’s normal sodium intake, and more measures would be more representative of the habitual sodium consumption of the individual. Nonetheless, the collection of a 24 h urine sample is a technique that is able to assess sodium consumption without bias, and is recognized as the gold standard procedure to assess sodium intake [31]. Nevertheless, even though the standard method for evaluating sodium intake is the 24 h urine collection, it may exhibit inaccuracies and collection errors [41]. To overcome this limitation, the samples were tested for creatinine coefficient to assure a valid urine sample [25]. 

Moreover, the utilization of a cross-sectional design restricts the ability to establish causal relationships between increased adherence to the MD and 24 h sodium excretion. Furthermore, our reliance on a 24 h recall questionnaire, designed primarily for short-term intake assessment, fails to account for fluctuations linked to different seasons. As a single day’s report may not accurately mirror habitual consumption, employing multiple recalls to depict an individual’s customary intake is a preferable approach. It is noteworthy, however, that our data collection approach encompassed a comprehensive range of insights, including portion sizes, constituents of mixed dishes, and specific commercial product brands. This meticulous approach ensured a complete characterization of consumption habits and dietary pattern [42]. Additionally, employing the 24 h recall questionnaire did not disrupt the dietary routines of the children, nor did it prompt them to alter their eating habits on account of the time-intensive aspect of documentation or heightened awareness of having a dietary evaluation. This methodology facilitated the assessment of their present eating patterns without inducing any modifications in their dietary conduct [43]. Yet, it is imperative to acknowledge that potential recall bias and indirect reporting might influence the accuracy of the dietary data collected, particularly considering that children’s self-reports of their dietary choices are susceptible to inaccuracies stemming from limited food knowledge and memory constraints [44]. However, opting for a 24-hour recall might be more favorable when assessing the typical dietary consumption of extensive sets of participants [45]. Moreover, it would be of substantial interest if an outcome concerning blood pressure could be established in relation to aMED and the levels of sodium excretion in urine. However, studying how a dietary pattern, such as the Mediterranean diet, relates to urinary sodium excretion holds the potential to contribute significantly to the body of scientific knowledge in this field and pave the way to further explore this subject: a seemingly healthier diet, such as the Mediterranean diet, might not always correspond to a reduced intake of sodium.

Our research exhibits some strengths that should be highlighted. To the best of our knowledge, this study is the first to explore the association between adherence to the Mediterranean dietary pattern, as attributed by the aMED score, and 24 h urinary sodium excretion. Additionally, as previously referred, our study utilized the 24 h urine collection to estimate sodium intake which allows to not induce bias: Parallelly, this method is regarded as the gold standard to assess sodium consumption. Also, this investigation encompassed a substantial total of individuals, and we meticulously considered significant potential confounding variables such as age, sex, parental education, overall energy intake, physical activity, and BMI. Lastly, opting for the alternate Mediterranean diet score offers a range of advantages owing to its comprehensive methodology, which effectively captures the interrelated components within the dietary regimen. In contrast to concentrating solely on isolated elements or nutritional factors, the aMED score encompasses a wide array of food components within the dietary pattern.

Finally, it is important to note that a review referred that the MD may have a lower significant effect on blood pressure compared to the DASH-diet because it is not strictly a low-salt dietary pattern [46]. Nonetheless, it is crucial to emphasize that the benefits of the Mediterranean diet expand outside the quantity of sodium. And the MD has demonstrated protective effects on airway inflammation [47], asthma [10], cancer [12], hypertension [7], and other cardiovascular diseases [11]. Further investigation is warranted to explore the association between sodium and the MD, and to uncover which are the principal contributors to sodium intake in this age group. 

## 5. Conclusions

Our study indicate that the MD dietary pattern is not associated with a lower urinary sodium excretion. Our results highlight the importance of taking concrete measures to reduce sodium content in food products. Additionally, there is a need to educate the population about the significance of incorporating less sodium in their cooking practices and reducing table salt usage during meals. As the complexities of dietary influences continue to unfold, a multifaceted approach that addresses sodium reduction through a collective commitment spanning dietary practices, food industry practices, and public awareness initiatives may be fundamental for reducing sodium intake.

## Figures and Tables

**Table 1:** Main characteristics of the study sample by median sodium urinary excretion values.

	Total, *n* = 295 (100%)	Sodium (≤2484 mg)	Sodium (>2484 mg)	*p*-Value
Age (years), mean ± SD	8.53 ± 0.73	8.45 ± 0.63	8.63 ± 0.82	0.043 *
Sex, *n* (%)				0.004 *
Female	138 (46.8%)	82 (55.0%)	56 (38.4%)	
Male	157 (53.2%)	67 (42.7%)	90 (57.3%)	
BMI				0.015 *
Non-overweight/obese (*p* < 85th)	226 (76.6%)	123 (82.6%)	103 (70.5%)	
Overweight/obese (*p* ≥ 85th)	69 (23.4%)	26 (17.4%)	43 (29.5%)	
aMED score, mean ± SD	2.66 ± 1.54	2.62 ± 1.52	2.69 ± 1.50	0.596
Vegetables, g, median (25th–75th)	107.02 ± 92.08	111.59 ± 96.60	102.37 ± 87.31	0.390
Fruits, g, mean ± SD	194.43 ± 168.61	203.76 ± 166.14	184.91 ± 171.14	0.338
Ratio MUFA to SFA	1.18 (0.91–1.47)	1.22 (0.91–1.52)	1.14 (0.89–1.40)	0.130
Fish (g), median (25th–75th)	0.00 (0.00–100.00)	0.00 (0.00–100.00)	0.00 (0.00–80.00)	0.553
Nuts(g), median (25th–75th)	0.00 (0.00–0.00)	0.00 (0.00–0.00)	0.00 (0.00–0.00)	0.436
Whole grains, (g), median (25th–75th)	0.00 (0.00–0.00)	0.00 (0.00–0.00)	0.00 (0.00–0.00)	0.510
Legumes, (g), median (25th–75th)	0.00 (0.00–14.52)	0.00 (0.00–0.00)	0.00 (0.00–33.38)	0.035 *
Red and processed meat, (g), median (25th–75th)	90.70 (30.0–159.9)	90.00 (34.85–154.15)	92.32 (20.00–161.53)	0.719
Total energy intake (kcal), median (25th–75th)	2223.72 (1889.76–2482.90)	2154.17 (1817.75–2410.48)	2224.12 (1923.87–2559.93)	0.043 *
Physical activity ^a^, *n* (%)				0.162
<2 timesx/week	128 (47.2%)	72 (56.3%)	56 (42.1%)	
2–3 times/week	105 (38.7%)	48 (34.8%)	57 (42.9%)	
≥4 times/week	38 (14.0%)	18 (13.0%)	20 (15.0%)	
Parental education ^b^, *n* (%)				0.233
<9 years	87 (35.1%)	39 (29.3%)	48 (41.7%)	
10–12 years	63 (25.4%)	41 (30.8%)	22 (19.1%)	
>12 years	98 (39.5%)	53 (54.1%)	45 (39.1%)	

Note: * stands for statically significant associations; Abbreviations: aMED: alternate Mediterranean score; MUFA: monounsaturated fatty acids; SFA: saturated fatty acids; %TEI: total energy intake. ^a^ <2 times/week, 2–3 times/week, or >4 times/week; ^b^ number of successfully completed years of formal schooling. Significant differences: α-value of less than 5% with a 95% confidence interval.

**Table 2:** Analysis of the association between the aMED score with urinary sodium excretion.

	aMED Score Crude Model, OR (95% CI)	*p*-Value	aMED Score Adjusted Model, OR (95% CI)	*p*-Value
Increased levels of excreted urinary sodium (>2484 mg)				
All participants	0.96 (0.83–1.12)	0.595	1.08 (0.91–1.28)	0.393

Note: Abbreviations: aOR: adjusted odds ratio; aMED: alternate Mediterranean score. Logistic regressions were adjusted to total energy intake, sex, age, parental education, physical activity, and BMI. Significant differences: α-value of less than 5% with a 95% confidence interval.

## Data Availability

The data that support the findings of this study will be made available by the authors upon reasonable request.
